# Effect of Kinins on the Hepatic Oxidative Stress in Mice Treated with a Methionine-Choline Deficient Diet

**DOI:** 10.3390/biomedicines11082199

**Published:** 2023-08-04

**Authors:** Mariana Silva Thomaz, Marcela Nascimento Sertorio, Marcos Leoni Gazarini, Daniel Araki Ribeiro, Luciana Pellegrini Pisani, Marcia Regina Nagaoka

**Affiliations:** Department of Biosciences, Instituto Saúde Sociedade, Universidade Federal de São Paulo, Santos 11015-020, SP, Brazil; msthomaz@unifesp.br (M.S.T.);

**Keywords:** liver, oxidative stress, steatosis, methionine-choline deficient diet, NASH

## Abstract

Non-alcoholic fatty liver is the leading cause of hepatic disease worldwide and ranges from simple steatosis to non-alcoholic steatohepatitis (NASH) due to cell injury, oxidative stress, and apoptosis. The kinins’ role in the liver has been studied in experimental fibrosis, partial hepatectomy, and ischemia-reperfusion and is related to cell death and regeneration. We investigated its role in experimental NASH induced by a methionine-choline deficient diet for 4 weeks. After that, liver perfusion was performed, and bradykinin (BK) or des-Arg^9^-BK was infused. Cell death was evaluated by cathepsin-B and caspase-3 activity and oxidative stress by catalase (CAT), glutathione S-transferase, and superoxide dismutase (SOD) activities, as well as malondialdehyde and carbonylated proteins. In control livers, DABK increased CAT activity, which was reversed by antagonist DALBK. In the NASH group, kinins tend to decrease antioxidant activity, with SOD activity being significantly reduced by BK and DABK. Malondialdehyde levels increased in all NASH groups, but carbonylated protein did not. DABK significantly decreased cathepsin-B in the NASH group, while caspase-3 was increased by BK in control animals. Our results suggest that B1R and/or B2R activation did not induce oxidative stress but affected the antioxidant system, reducing SOD in the NASH group.

## 1. Introduction

Non-alcoholic fatty liver disease (NAFLD) is the leading cause of liver disease worldwide and affects 25% of the adult population [1]. NAFLD ranges from simple steatosis to a more severe form of liver disease: non-alcoholic steatohepatitis (NASH) [2].

NASH is characterized by steatosis, lobular inflammation, and hepatocyte ballooning degeneration and may present a variable degree of fibrosis degree [3]. Initially, NASH development was proposed by the “two-hit” theory, in which the first hit was steatosis and the second hit was oxidative stress [4]. However, considering the disease’s complexity, the “two-hit” theory was insufficient to explain its development, and the “multiple-hit” theory is now more accepted: the first hits are insulin resistance and metabolic syndrome, and the other hits are a combination of inflammation, oxidative stress, mitochondrial dysfunction, hepatic lipid peroxidation, apoptosis, and fibrosis [5,6,7].

Insulin resistance stimulates hepatic de novo lipogenesis and impairs the inhibition of lipolysis in adipose tissue, increasing the levels of free fatty acids (FFA) [7], which will be captured by the liver and stored as lipid vesicles, culminating in steatosis. Lipotoxicity-induced liver injury generates a cascade of alterations, such as activation of hepatic stellate cells and the increased intracellular signaling of inflammation and apoptosis, besides the increase in reactive species of oxygen (ROS) [8,9]. Oxidative stress, which is an imbalance in ROS production with a deficient antioxidant system defense capacity such as catalase (CAT), superoxide dismutase (SOD), and glutathione peroxidase (GPx), has a pivotal role in the development and progression of NAFLD [10,11,12,13,14].

During the progression of NAFLD, the main sources of ROS in the liver are mitochondrial dysfunction caused by increased mitochondrial β-oxidation, the influx of FFAs into the liver, and endoplasmic reticulum stress [15]. As a consequence, ROS can lead to cell damage by interacting with fatty acids in the cell membrane, initiating the process of lipid peroxidation [16]. Malondialdehyde (MDA), one of the products of lipid peroxidation, can stimulate the release of chemokines and cytokines and the activation of hepatic stellate cells, contributing to inflammation, fibrosis, and apoptosis. Furthermore, MDA can increase ROS production, resulting in a cyclic mechanism of ROS-MDA [12].

The important role of oxidative stress in the development of NASH can be evaluated in clinical and animal models of NAFLD/NASH [5,17]. Among animal models of NASH, the methionine-choline-deficient (MCD) diet appears to mimic oxidative stress biomarkers found in clinical disease, leading to an increase in MDA levels and a decrease in the activity of antioxidant enzymes, such as CAT, SOD, and GPx [5]. Furthermore, the MCD diet replicates the histopathological features observed in NASH [17]. So, the MCD diet is the best choice for the study of oxidative stress in the murine model of NASH [18]. Inflammation and related signaling pathways, as one of the components of NASH, are extensively studied in an attempt to elucidate its pathogenesis [19,20], and the involvement of the kallikrein–kinin system in this pathology can be speculated. Kinins are a group of vasoactive peptides that participate in the inflammatory process, such as vasodilation, increased vascular permeability, and nitric oxide production [21]. The pharmacological effects of kinins are mediated by two G protein-coupled receptors: the B1 receptor (B1R) and the B2 receptor (B2R) [22,23]. B2R is a constitutive receptor and is activated mainly by bradykinin (BK), while B1R is induced by inflammation and tissue injury and is activated by des-Arg^9^-BK (DABK), a metabolite of BK formed by the action of kininases [24,25].

The liver participates in the modulation of the kallikrein–kinin system [26], and the involvement of the kinin system has been described in different experimental models of liver diseases, such as fibrosis, liver regeneration, and ischemia–reperfusion [27,28]. Furthermore, kinins have been reported to reduce oxidative stress and tissue damage in the kidney and heart [29]. In this context, this study aimed to investigate the effects of kinins in the liver of MCD diet-fed mice, looking for a better comprehension of the pathophysiology of NASH.

## 2. Materials and Methods

### 2.1. Animals and Experimental Design

Six-week-old male Swiss mice were obtained from the Federal University of São Paulo. The experiments were performed following the Guide for the Care and Use of Laboratory Animals [30] and approved by a local committee (protocol number 2137270219). The animals were kept at a constant temperature of 22 ± 2 °C on a 12 h light–dark cycle with ad libitum access to food and water. The animals were randomly allocated into two major experimental groups according to the diet obtained from Rhoster^®^ (Araçoiaba da Serra, Brazil). The control group received a control diet (AIN-76, RH19522), and the NASH group received a methionine-choline deficient diet (MCD, RH19524E) for 4 weeks. Throughout the experimental period, the animals were weighed twice a week, and feed consumption was evaluated daily.

### 2.2. In Situ Liver Perfusion (Experimental Design)

The animals were anesthetized with urethane (1.3 g/kg; intraperitoneal), weighed, and prepared for laparotomy. Blood was collected by cardiac puncture with 0.1 M sodium citrate, and the plasma was separated for later biochemical analysis. The portal vein (inflow cannula) and thoracic inferior vena cava (outflow cannula) were cannulated, and the livers were exsanguinated at a constant flow of 1 mL/min.

To evaluate the acute effect of kinins on oxidative stress and cell death pathway, the agonist of B1R (DABK, 23 nmol) or B2R (BK, 23 nmol) was infused with 1 mL of Krebs–Henseleit bicarbonate solution (Krebs solution); the same volume of NaCl 0.15 M was used as a control of agonists addition. To analyze the specific effect, the antagonist of B1R (DALBK, 0.7 μmol/L) or B2R (HOE-140, 0.7 μmol/L) was infused previously to the respective agonist addition [31].

Finally, the livers were weighed, a fragment was fixed in 4.5% formaldehyde for histological analysis, and another was frozen in liquid nitrogen and stored at 80 °C for later analysis.

### 2.3. Biochemical Analysis

#### 2.3.1. Glucose

Glucose was measured, through blood collected from the caudal vein, using a glycosometer (Roche Diagnostics, Basel, Switzerland).

#### 2.3.2. Cholesterol and Triglycerides

Plasma levels of cholesterol and triglycerides were measured using Labtest Diagnostica kits (Lagoa Santa, Brazil), following the manufacturer’s instructions.

#### 2.3.3. Aminotransferases

Serum activity of aspartate aminotransferase (AST) and alanine aminotransferase (ALT) were measured using Labtest Diagnostica kits (Lagoa Santa, Brazil), following the manufacturer’s instructions.

### 2.4. Histological Analysis

Images were acquired using the AxioImager A2 microscope with an AxioCam MRc camera and analyzed using AxioVision 4.8 software (Zeiss, Rostock, Germany). The specimen was evaluated according to the previously reported NAS (NAFLD Activity Score) score [32,33,34,35].

### 2.5. Cell Death Pathway Analysis by Cathepsin B and Caspase-3 Activity

Liver samples were homogenized with 100 mM Trizma-Base pH 7.5, containing 20 mM EDTA, 100 mM Na_4_P_2_O_7_, 100 mM NaF, 10 mM Na_3_VO_4_, 2 mM PMSF, and 0.1 mg/mL protease inhibitor (Sigma-Aldrich, Saint Louis, MO, USA); after homogenization, Triton-X was added into the samples. Samples were centrifuged at 10,000× *g*, at 4 °C, for 10 min. The supernatant was separated, and the protein concentration was measured with a Pierce BCA Protein kit (Thermo Fisher Scientific, Waltham, MA, USA).

Cathepsin B activity was measured by incubating liver homogenates with 0.58 µM 2-amino benzoyl-Gly-Ile-Val-Arg-Ala-Lys (2,4-dinitrophenol) (Abz-GIVRAK(Dnp)-OH, Aminotech, Diadema, Brazil) in 75 mM Tris, 25 mM MES, 25 mM acetic acid, 25 mM glycine, 2 mM EDTA, and 2 mM dithiothreitol (pH 4.5) in a 96-well plate at 37 °C for 10 min. Fluorescence was measured using a spectrophotometer (F-7000; Hitachi, Tokyo, Japan) at an excitation wavelength of 320 nm and an emission wavelength of 420 nm. Results are expressed as arbitrary fluorometric units per minute per microgram of protein (AFU·min^−1^·µg^−1^) [36].

For caspase-3 activity, liver homogenates were incubated with 200 µM N-Acetyl-Asp-Glu-Val-Asp-7-amido-4-Methylcoumarin (Ac-DEVD-AMC, Sigma-Aldrich, USA) in 50 mM HEPES, 100 mM NaCl, 0.1% CHAPS, 1 mM EDTA, and 2 mM dithiothreitol (pH 7.4) in a 96-well plate at 37 °C for 60 min in a Hitachi F-7000 spectrofluorimeter (λex = 380 nm and λem = 460 nm). Results are expressed as arbitrary fluorometric units per minute per milligram of protein (AFU·min^−1^·mg^−1^) [37,38].

### 2.6. Oxidative Stress Analysis

Liver samples were homogenized with phosphate buffer pH 7.4, containing 1.4 mM KH_2_PO_4_, 2.65 mM K_2_HPO_4_, and 1 mM EDTA. Samples were centrifuged at 10,000× *g*, at 4 °C, for 10 min. The supernatant was used for the analysis of catalase (CAT), superoxide dismutase (SOD), glutathione S-Transferase (GST), and malondialdehyde (MDA), while the pellet was used for the analysis of protein carbonyl (PC). The protein concentration of homogenates was measured with a Pierce BCA Protein kit (Thermo Fisher Scientific, USA).

#### 2.6.1. Catalase Activity

Liver homogenates were incubated with 20 mM H_2_O_2_. To interrupt the reaction, 4% ammonium molybdate was used. Through H_2_O_2_ decomposition, the CAT activity was measured [39]. Results were expressed as UCAT/min/mg protein·g liver.

#### 2.6.2. Glutathione S-Transferase Activity

Liver homogenates were incubated with phosphate buffer, pH 7.0, 0.1 M 1-chloro-2,4-dinitrobenzene (CDNB), and 100 mM glutathione (GSH). The GST activity was measured by the conjugation of glutathione tiol groups to 1-chloro-2,4-dinitrobenzene [40]. Results were expressed as μmol/min/g/mg protein·g liver.

#### 2.6.3. Superoxide Dismutase Activity

Liver homogenates were incubated with phosphate buffer, pH 7.0, 1.25 mM 3-(4,5-dimethyl-2-thiazolyl)-2,5-diphenyl-2H-tetrazolium bromide (MTT), and 10 mM pyrogallol. The SOD activity was measured through the reaction of superoxide and hydrogen peroxide catalyzed by this enzyme. Results were expressed as USOD/mg protein·g liver [41].

#### 2.6.4. Levels of Malondialdehyde

Liver homogenates were incubated with the thiobarbituric acid solution containing 15% trichloroacetic acid (TCA), 0.375% thiobarbituric acid (TBA), and 0.25 M hydrochloric acid (HCl). The levels of MDA were measured by the formation of thiobarbituric acid reactive substances [42]. Results were expressed as μM/mg protein·g liver.

#### 2.6.5. Levels of Carbonyl Protein

Liver pellets were resuspended with phosphate buffer pH 7.4 and then incubated with 10 mM 2,4-dinitrophenylhydrazine (DNPH), and after 30 min, 10% TCA was added. The levels of carbonyl protein were measured by the derivatization of carbonyl groups with 2,4- dinitrophenylhydrazine, leading to the formation of dinitrophenyl hydrazones [43]. The molar extinction coefficient of carbonyl was used to calculate the results, which were expressed as nmol/mL/mg protein·g liver.

### 2.7. Statistical Analysis

Sample normality was evaluated using Shapiro–Wilk and Kolgomorov–Sminorv tests, and Z-score transformation was used for data normalization when necessary. Samples were analyzed by *t*-test or ANOVA followed by post hoc Tukey. Results were considered statistically significant when *p* < 0.05. The statistical analysis was performed using IBM SPSS 22 and GraphPad Prism 8 software.

## 3. Results

### 3.1. Experimental Model

#### 3.1.1. Quantification of Body Weight and Diet Consumption

**Figure 1:**
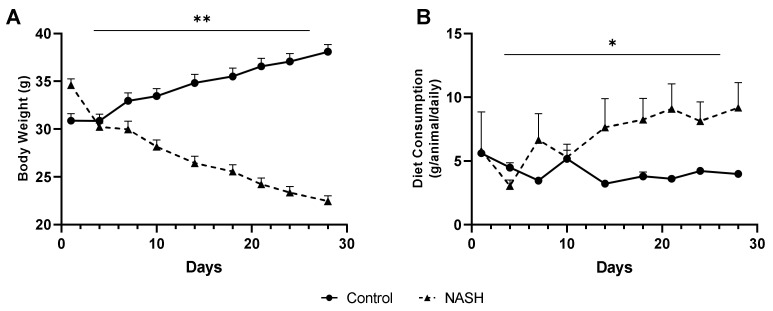
Quantification of body weight (g) (**A**) and diet consumption (g/animal/daily) (**B**) of mice submitted to AIN-76 (control group) or MCD diet (NASH group) for 4 weeks. Data expressed as mean ± SEM. N = 25/group. *t*-test: (**A**) t = 3.373; ** *p* = 0.009; (**B**) t = 3.351; * *p* = 0.010.

Figure 1 shows the body weight (A) and diet consumption (B) of mice submitted to the AIN-76 diet (control group) or MCD (NASH group) over 4 weeks. During this period, the animals in the NASH group showed weight loss (~12 g) compared to the weight gain (~7 g) of the control animals (t = 3.373; *p* = 0.009) (Figure 1A). Diet consumption was constant in the control group, in contrast to the increase after 10 days in the NASH group. Considering the entire induction time, the average diet intake (g/day/animal) was higher in the NASH group (7.0 ± 0.7) compared to the control (4.2 ± 0.3) (t = 3.351; *p* = 0.010) (Figure 1B).

#### 3.1.2. Descriptive Parameters

Table 1 presents the descriptive parameters analyzed: final body weight, liver weight, liver index, blood glucose, cholesterol, triglycerides, AST, and ALT.

Comparing the NASH group in relation to the control, the final body weight (g) in the NASH group was significantly (t = 18.23; *p* < 0.0001) lower (40%). Likewise, liver weight (g) was significantly (t = 6.71; *p* < 0.0001) lower (30%) in the NASH group. The liver index (liver/final body weight) was higher (t = 3.456; *p* = 0.0012) in the NASH group.

Both glycemia and serum cholesterol were significantly (*p* < 0.0001) lower in the NASH group compared to the control group (t = 9.778 for blood glucose, t = 4.91; for cholesterol). There were no differences in serum triglyceride levels between the experimental groups. ALT and AST aminotransferase levels increased by 2.7 (t = 4.812; *p* < 0.0001) and 1.4 times (t = 2.391; *p* = 0.021), respectively, in the NASH group compared to the control group.

### 3.2. Histopathological Analysis

**Figure 2:**
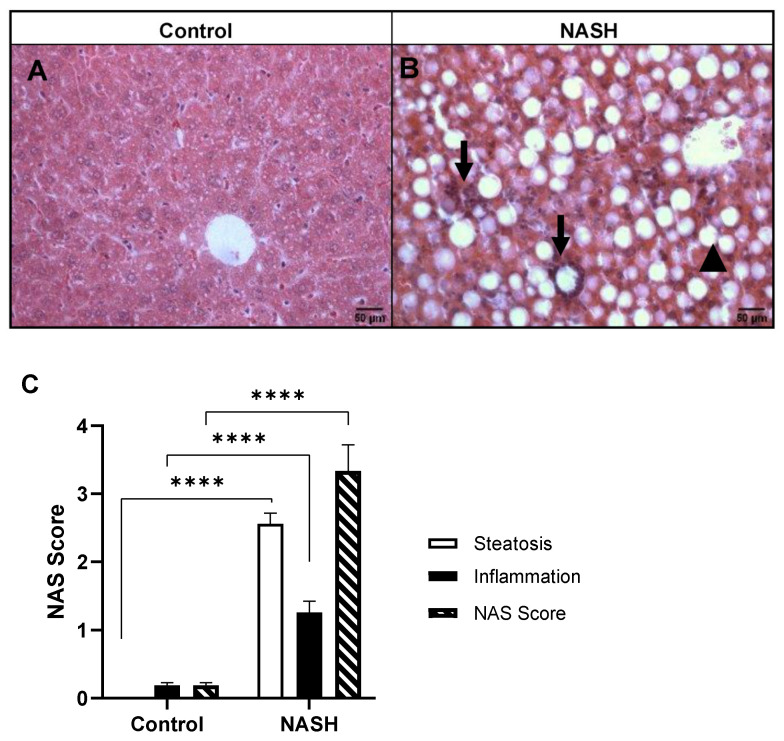
Representative photomicrography of hematoxylin-eosin stained liver from mice submitted to AIN-76 (control group; (**A**)) or methionine-choline deficient diet (NASH group; (**B**)). Black arrows indicate inflammatory focus, and black arrowheads indicate macrovesicular steatosis. Scale bar 50 μm. Hepatic steatosis score (**C**) for AIN-76- and MCD-fed mice for 4 weeks. Data expressed as mean ± SEM. N = 25/group. *t*-test: (**C**) steatosis: t = 16.66, **** *p* < 0.0001; inflammation: t = 10.92, **** *p* < 0.0001; NAS score: t = 16.94, **** *p* < 0.0001.

Figure 2 shows representative photomicrographs of liver sections stained with HE from mice submitted to the AIN-76 (control group) or MCD (NASH group) diet. In the control group (Figure 2A), the hepatic architecture was maintained, while the NASH group (Figure 2B) presented lipid vesicles (arrowheads) and inflammatory infiltrate (arrows), characterizing steatohepatitis. However, ballooning of hepatocytes was not found in all analyzed liver sections.

Figure 2C presents the scores for steatosis and inflammatory infiltrate, as well as the NAS. The NASH group has higher scores (*p* < 0.0001) for steatosis (t = 16.66), inflammatory infiltrate (t = 10.92), and NAS (t = 16.94) compared to the control group.

### 3.3. Oxidative Stress Analysis

**Figure 3:**
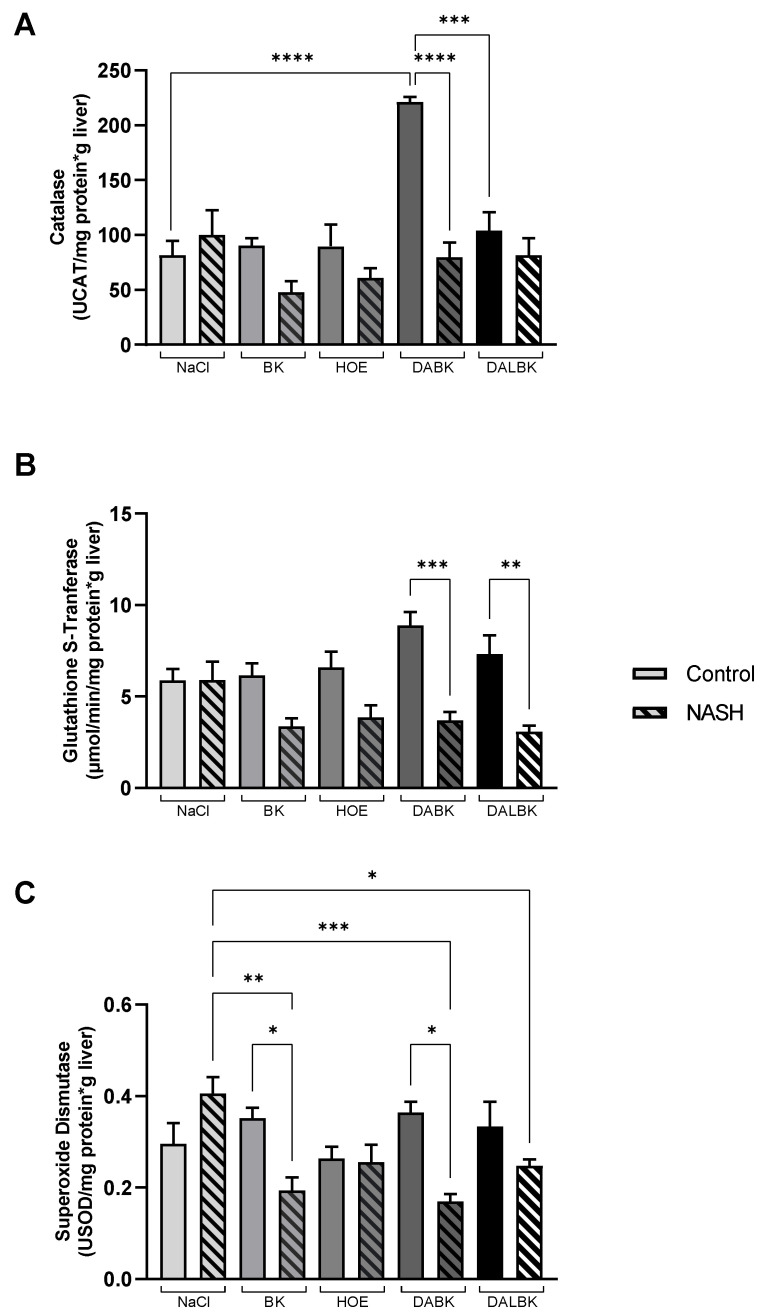
Measurement of total hepatic activity of the antioxidant enzymes. Catalase (UCAT/min/mg protein·g liver) (**A**), glutathione S-transferase (μmol/min/g/mg protein·g liver) (**B**), and superoxide dismutase (USOD/mg protein·g liver) (**C**) from mice fed with AIN-76 (control group) or MCD diet (NASH group), in the presence of kinin receptor agonists (BK, DABK) or antagonists (HOE, DALBK). BK: bradykinin (B2R agonist); DABK: des-Arg^9^-BK (B1R agonist); DALBK: des-Arg-Leu-BK (B1R antagonist); HOE: HOE-140 (B2R antagonist). Data expressed as mean ± SEM. N = 5/group. ANOVA post hoc Tukey: (**A**) F = 9.047, **** *p* < 0.0001, *** *p* < 0.001; (**B**) F = 7.229, *** *p* < 0.001, ** *p* < 0.01; (**C**) F = 5.082, *** *p* < 0.001, ** *p* < 0.01, * *p* < 0.05.

**Figure 4:**
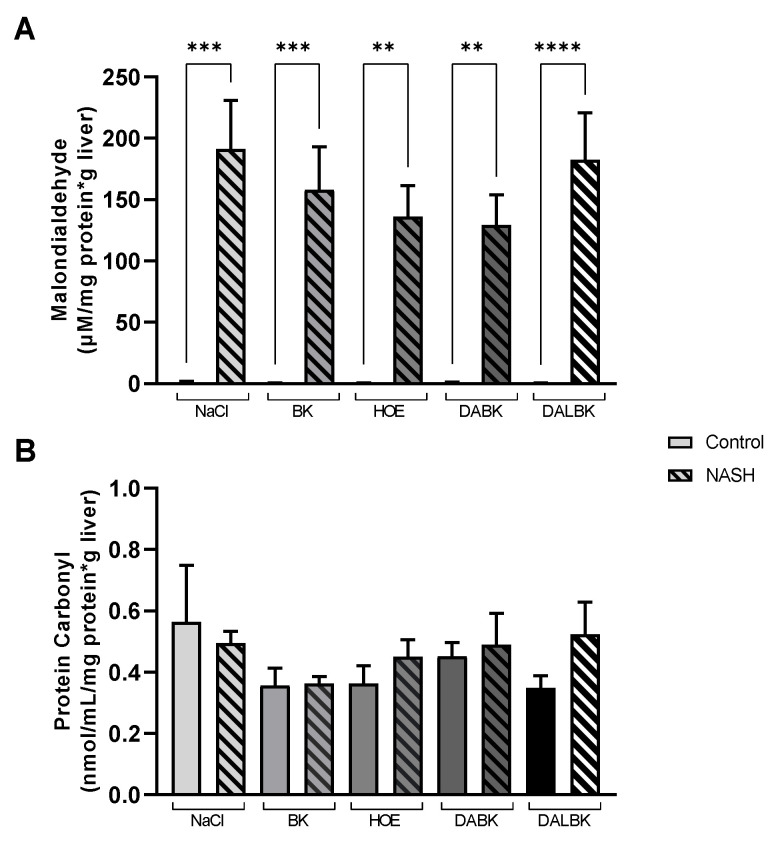
Measurement of the total hepatic level of malondialdehyde (μM/mg protein·g liver) (**A**) and protein carbonyl (nmol/mL/mg protein·g liver) (**B**) of mice fed with AIN-76 (control group) or MCD diet (NASH group), in the presence of kinin receptor agonists (BK, DABK) or antagonists (HOE, DALBK). BK: bradykinin (B2R agonist); DABK: des-Arg^9^-BK (B1R agonist); DALBK: des-Arg-Leu-BK (B1R antagonist); HOE: HOE-140 (B2R antagonist). Data expressed as mean ± SEM. N = 5/group. ANOVA: (**A**) F = 14.97, *p* < 0.0001, post hoc Tukey: **** *p* < 0.0001, *** *p* < 0.001, ** *p* < 0.01; (**B**) *p* = 0.6094.

To investigate the effect of kinins on oxidative stress in NASH, the activities of the antioxidant enzymes catalase (CAT), glutathione S-transferase (GST), and superoxide dismutase (SOD) were analyzed (Figure 3), in addition to lipid peroxidation markers such as malondialdehyde and protein oxidation as carbonyl protein (Figure 4).

The hepatic catalase activity (UCAT/mg protein x g liver) increased significantly in the control group when DABK (221 ± 5) was infused compared to NaCl infusion (82 ± 13; *p* < 0.0001) and DALBK (104 ± 17; *p* < 0.001) (Figure 3A). In the NASH group, DABK infusion decreased (*p* < 0.0001) catalase activity (80 ± 13) when compared to the control/DABK. However, hepatic glutathione S-transferase activity (µmol/min/g protein · g liver) decreased in all NASH groups in the presence of kinin receptor agonists or antagonists (Figure 3B). However, only in the NASH group, infused DABK (3.7 ± 0.5) or DALBK (3.1 ± 0.3) significantly decrease GST activity compared to their respective controls (DABK: 8.9 ± 0.7, *p* < 0.001; DALBK: 7.3 ± 1.0, *p* < 0.01).

The hepatic superoxide dismutase activity (USOD/mg protein x g liver) was lower in the NASH groups when BK (0.19 ± 0.03; *p* < 0.01) or DABK (0.17 ± 0.02; *p* < 0.001) were infused compared to the respective NaCl group (0.41 ± 0.04) and to the respective control groups with BK (0.35 ± 0.02; *p* < 0.05) or DABK infusion (0.36 ± 0.02; *p* < 0.05) (Figure 3C).

Hepatic malondialdehyde levels (µmol/g protein · g liver) increased (*p* < 0.0001) in all NASH groups, regardless of which substance was infused when compared to their controls (Figure 4A). No difference was observed in the total hepatic levels of protein carbonyl between the experimental groups studied (Figure 4B).

### 3.4. Cell Death Pathway Enzymes

**Figure 5:**
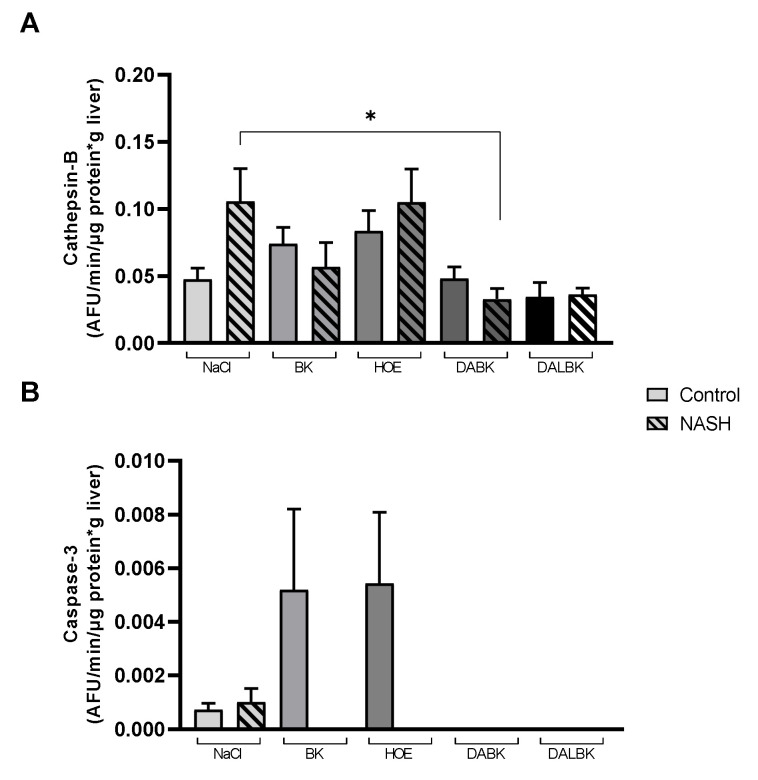
Measurement of total hepatic enzymatic activity (AFU/min/mg protein) of cathepsin B (**A**) and caspase-3 (**B**) from liver homogenates of mice fed with AIN-76 (control group) and MCD diet (NASH group) in the presence of kinin receptors agonists (BK, DABK) or antagonists (HOE, DALBK). BK: bradykinin (B2R agonist); DABK: des-Arg^9^-BK (B1R agonist); DALBK: des-Arg-Leu-BK (B1R antagonist); HOE: HOE-140 (B2R antagonist). Data expressed as mean ± SEM. N = 5/group. ANOVA: (**A**) F = 3.506; *p* = 0.0028, post hoc Tukey: * *p* < 0.05.

As ballooning can be a consequence of apoptosis or trigger it [44] and as ROS can be one of the mediators of cell death signaling [45], we evaluated apoptosis through the hepatic enzymatic activities of cathepsin-B and caspase-3 (Figure 5).

Total cathepsin-B activity decreased in the NASH group with DABK infusion compared to the respective NaCl group (*p* < 0.05) (Figure 5A). Regarding caspase-3 activity, there was very low activity in all studied experimental groups (Figure 5B); a positive control sample was used to confirm that the assay was working, and its caspase-3 activity (AFU/min/µm protein) was high (0.664) compared to the highest experimental data from some groups (0.006).

## 4. Discussion

This study aimed to evaluate the effect of kinins on oxidative stress in an experimental model of non-alcoholic steatohepatitis (NASH).

Non-alcoholic steatohepatitis (NASH) is a severe form of NAFLD, and it can progress to fibrosis, cirrhosis, and hepatocellular carcinoma (HCC) [6]. The worldwide prevalence of NASH is estimated from 1.5% up to 6.5%, and the disease is associated with diabetes, dyslipidemia, hypertriglyceridemia, and metabolic syndrome [1,46]. A 20% increase in NASH cases in the US is expected by 2030 [46], and this is associated with increased obesity, diabetes, and aging [47]. Considering that NASH is a public health matter, a better understanding of its physiopathology is essential. Although there are no FDA-approved drugs for the treatment of NASH, some signaling pathways have been described [19,20], including perspectives for treatment [48,49]. As far as we know, no work has been reported on the involvement of kinins in steatosis. However, there is evidence of a role for kinins in oxidative stress, which is one of the main events in NASH. Thus, we evaluated the effect of kinins on oxidative stress and cell death resulting from the development of NASH.

NASH was successfully induced by a methionine-choline-deficient (MCD) diet for 4 weeks, which is a well-established model that leads to histopathological changes such as steatosis, inflammatory infiltration, and ballooning [50,51,52,53]. As early as 10 days into a diet with MCD, steatosis with inflammatory infiltrate appeared in the mice’s liver [54,55].

The weight loss observed over the experimental period is characteristic of the MCD diet and has been described after 2 [56], 4 [52], and 8 weeks [57]. It seems to be related to hypermetabolism caused by the MCD diet due to an increase in body energy expenditure, a higher rate of fatty acid oxidation, and mitochondrial uncoupling. Despite the weight loss, the MCD diet group had a higher diet consumption, suggesting an increase in body energy expenditure. Interestingly, even with lower body and liver weight, the liver index was higher in the NASH group, indicating steatosis [58].

The MCD diet is high in sucrose (40%) and moderate fat (10%) and lacks methionine and choline [59]. The lack of methionine alters the synthesis of adenosylmethionine (SAM) and glutathione (GSH) by reducing their levels [60]. SAM is responsible for the methylation of proteins and DNA [61]; meanwhile, GSH is a potent antioxidant [62]. The lack of choline alters the synthesis of phosphatidylcholine, which is required for VLDL secretion [63] and an essential component of cell membranes [64]. Thus, the absence of methionine contributes to hepatocellular injury, inflammation, oxidative stress, and choline deficiency in steatosis [60]. The metabolic parameters showed a decrease in serum cholesterol and glycemia in NASH, and this has already been reported in MCD-fed mice [65]. Downregulation of genes involved in cholesterol biosynthesis, such as HMG-CoA synthase 1 and HMG-CoA reductase, as well as downregulation of glucose metabolism genes, such as GLUT2, glucokinase, phosphofructokinase, glycogen synthase, and phosphorylase, in MCD-fed mice [66] can explain the metabolic profile found in our results. Furthermore, reduced serum levels of cholesterol and blood glucose seem to be related to a higher diet intake, and this metabolic profile should further stimulate diet intake [58].

The MCD diet led to hepatic steatosis with inflammatory infiltrate but without hepatocyte ballooning. Despite ballooning being considered a typical feature of NASH [3,44], the lack of ballooning had already been reported [67]. As apoptosis could be a consequence of ballooning or a trigger for ballooning formation [44], the apoptosis pathway was evaluated by cathepsin-B and caspase-3 activities. Hepatic cathepsin-B activity was present in all groups, but in the NASH group, it decreased in the presence of DABK. In vitro, bovine, human, and rat plasma high-molecular and low-molecular-mass kininogens can inhibit rat liver cathepsin-B activity, and kinin-free proteins have stronger inhibitory activity [68]. In the used liver perfusion model, the blood is replaced by Krebs solution, so only DABK activation is being studied, and there was no influence of kininogens, so DABK did not directly inhibit cathepsin B, but B1R activation by DABK may produce an inhibitory stimulus for cathepsin B.

Surprisingly, there was no activity of caspase-3 in all studied groups. Cathepsin-B can activate apoptosis through caspase-3 but also stimulates NF-κB translocation and inflammatory response [69,70]. So, we hypothesized that the hepatic activity of cathepsin-B is lower to activate apoptosis via caspase-3, but it can contribute to NF-κB translocation. Moreover, the lack of caspase-3 may indicate a low level of apoptosis and be related to the absence of ballooning in the histopathological analysis since apoptosis could be a consequence of ballooning or a trigger for ballooning formation [44].

Considering that oxidative stress is pivotal in mediating the development of steatohepatitis in the MCD diet model, the MCD diet-induced NASH is a suitable choice to study oxidative stress biomarkers. Thus, the effect of kinins on oxidative stress in this experimental model of NASH was evaluated by the activity of antioxidant enzymes and markers of lipid peroxidation and protein oxidation.

Catalase (CAT) activity in control mice increased in the presence of DABK, and when its antagonist was added, activity decreased to normal levels, suggesting a specific effect of B1R activation. Interestingly, this effect was not observed in NASH. Although the MCD diet reduced GSH levels [60], there was no effect on glutathione S-transferase (GST) activity, as its activity was the same in the control and NASH groups in the absence of kinins. It has been shown that B1R activation can extend oxidative stress, increasing the production of ROS and antioxidant enzymes such as CAT and SOD in thoracic aorta isolated from rats fed with glucose for 12 weeks, an experimental model of insulin resistance [71]. Later, it was shown that B1R activation contributes to insulin resistance by promoting systemic inflammation [72]. Thus, in normal liver, this stimulatory effect on antioxidant enzymes by B1R activation remains; however, there was a lack of this effect in NASH, and oxidative stress was probably maintained by increased production of ROS, which stimulated the inflammation observed in NASH.

Regarding superoxide dismutase, the addition of BK to the experimental NASH decreased SOD activity. In fact, increased serum SOD activity was reported in streptozotocin-induced diabetes [73] and in a model of H_2_O_2_-induced senescence in isolated cardiomyocytes both via B2R activation and NO release, which promotes vasodilation [74]. In contrast, in the hepatic portal system, BK induces NO-mediated vasoconstriction [75], which may explain the decrease in SOD activity in the liver in the NASH model. Furthermore, hepatic SOD activity decreased when DABK was administered into NASH and interestingly, when the B1R antagonist was added, a tendency to normalize was observed, suggesting a role for B1R activation in SOD activity. Indeed, B1R activation increases oxidative stress related to O_2_-levels [71], and our results suggest that B1R may also contribute to oxidative stress by decreasing SOD activity.

Lipid peroxidation, as assessed by malondialdehyde levels, increased in NASH, which is a well-established consequence of the MCD diet, with an increase described as early as 10 days in mice fed MCD and remaining elevated after 5 weeks [54]. Kinins, in turn, did not modulate MDA levels in this experimental model, suggesting that the effects of kinins on oxidative stress exacerbation in our model are due to a reduction in antioxidant enzyme activity but not related to increased lipid peroxidation.

In the present study, it was shown that kinins can modulate the activity of antioxidant enzymes, mainly SOD, and the activity of cathepsin-B in the experimental NASH. It is known that kinins can modulate oxidative stress by reducing their levels in the kidney and heart [29], but they can also increase oxidative stress through B1R activation, contributing to perpetuating inflammation [71,72]. On the other hand, so far, the relation between the activation of kinin receptors and cathepsin-B levels has not been described.

A limitation of the study is the acute infusion of the kinins in the liver perfusion. The agonists of B1R and B2R had significant effects, but the antagonists of the receptors seemed to need more time to action to block their receptors and reverse the effects caused by the agonists.

Considering that hepatic alterations are the main features of NASH, in the current study, the aim was to study the acute effect of kinins in the liver to provide a better understanding of its local effects. Moreover, the systemic effect of kinins and their relationship with NASH is our goal for future studies.

In conclusion, under normal conditions, B1R activation promotes an increase in catalase activity, which is not maintained in NASH. On the other hand, activation of B1R or B2R decreased SOD activity in NASH, suggesting that kinins contribute to the maintenance of oxidative stress and disease progression. These results suggest the role of intracellular signaling of kinins in the activity of antioxidant enzymes.

## 5. Conclusions

Under normal conditions, B1R activation promotes an increase in catalase activity, which is not maintained in NASH. On the other hand, activation of B1R or B2R decreased SOD activity in NASH, suggesting that kinins contribute to the maintenance of oxidative stress and disease progression. These results suggest the role of intracellular signaling of kinins in the activity of antioxidant enzymes.

## Figures and Tables

**Table 1:** Descriptive parameters of animals submitted to AIN-76 (control group) or MCD diet (NASH group).

	Control	NASH	*p*
Final Body Weight (g)	37.7 ± 0.8	22.0 ± 0.5	****
Liver Weight (g)	1.81 ± 0.04	1.28 ± 0.07	****
Liver Index (%)	4.8 ± 0.1	5.8 ± 0.3	**
Glycemia (mg/dL)	206 ± 10	103 ± 5	****
Cholesterol (mg/dL)	120 ± 4	89 ± 5	****
Triglycerides (mg/dL)	122 ± 4	123 ± 8	
ALT (U/L)	153 ± 20	414 ± 48	****
AST (U/L)	303 ± 28	433 ± 44	*

Legend: Data expressed as mean ± SEM. N = 25/group. *t*-test: compared do the control group: * *p* < 0.05, ** *p* < 0.01, **** *p* < 0.0001.

## Data Availability

The data presented in this study are available on request from the corresponding author.
